# Hit hard and early: analysing the effects of high-dose methylprednisolone on nailfold capillary changes and biomarkers in very early systemic sclerosis: study protocol for a 12-week randomised controlled trial

**DOI:** 10.1186/s13063-018-2798-x

**Published:** 2018-08-22

**Authors:** Wieneke M. T. van den Hombergh, Brigit E. Kersten, Hanneke K. A. Knaapen-Hans, Rogier M. Thurlings, Peter M. van der Kraan, Frank H. J. van den Hoogen, Jaap Fransen, Madelon C. Vonk

**Affiliations:** 1 Department of Rheumatology, Radboud University Medical Center, Geert Grooteplein Zuid 10, 6525 GA Nijmegen, The Netherlands; 2 Experimental Rheumatology, Radboud University Medical Center, Geert Grooteplein Zuid 10, 6525 GA Nijmegen, The Netherlands

**Keywords:** Systemic sclerosis, Methylprednisolone, Randomised controlled trial, Very early diagnosis of systemic sclerosis, Nailfold capillaroscopy

## Abstract

**Background:**

Mounting evidence indicates that inflammatory mechanisms drive systemic sclerosis (SSc) vasculopathy and fibrosis, especially early in the disease. Therefore, patients with very early SSc could benefit from early treatments targeting inflammation. Glucocorticoids are among the most potent anti-inflammatory and immunosuppressive agents. Several studies have demonstrated a mixed response to treatment with glucocorticoids in SSc, probably because it is seldom initiated at very early stages of the disease. We hypothesise that by inhibiting the inflammatory process driving SSc disease progression, glucocorticoid treatments will induce remission in patients with very early SSc.

**Methods/design:**

This study is a 12-week, randomised, double-blind, placebo-controlled trial analysing the effects of high-dose intravenous methylprednisolone in very early SSc. Thirty patients who fulfil the criteria for very early SSc will be randomly assigned in a 2:1 ratio to receive either intravenous methylprednisolone or a placebo on three consecutive days over three consecutive months. In this study, the primary endpoint will be the change in capillary density between the baseline and after 12 weeks of treatment. The secondary outcomes of this study are a change in selected biomarkers, other changes in the nailfold capillaries, signs of established SSc and changes in physical function, general health and utilities, as reported through questionnaires.

**Discussion:**

This trial is the first aiming to treat very early SSc and is promising because it targets the very early stages of the disease process by using an inexpensive and relatively safe treatment known to be highly effective against inflammation. The use of vasculopathy and inflammatory biomarkers as well as clinical signs and symptoms as the endpoints in our study enables us to meet the patient need for markers of disease activity. If it is possible to prevent clinically significant disease in patients with very early SSc by using a safe treatment, this will cause a paradigm shift in scleroderma care and research.

**Trial registration:**

ClinicalTrials.gov Identifier: NCT03059979. Registered on 20 February 2017.

**Electronic supplementary material:**

The online version of this article (10.1186/s13063-018-2798-x) contains supplementary material, which is available to authorized users.

## Background

In systemic sclerosis (SSc), the extent of organ involvement largely determines patient quality of life and disease burden. Organ involvement often occurs in the first three years of the disease and is tightly linked to an increase in mortality, decreasing the 5-year survival rate to 84% to 91%. To date, no treatment is available to cure SSc and its aetiology is unknown [1]. The major pathogenic features of SSc are a dysregulation of the immune system, dysfunction of the vasculature, and the activation of fibroblasts and other resident cells. Several studies have indicated that inflammatory mechanisms drive SSc vasculopathy and fibrosis, especially at the early stages of the disease [2–7].

In addition, it is likely that SSc shares an autoimmune inflammatory basis with other systemic autoimmune diseases such as lupus nephritis [8]. Glucocorticoids, some of the most potent anti-inflammatory and immunosuppressive agents, can induce remission in systemic autoimmune diseases, including lupus nephritis and dermatomyositis, and have an immediate and profound effect on the trafficking of leucocytes [9–11]. Furthermore, glucocorticoids affect both T- and B-lymphocytes.

A systematic review on the use of glucocorticoids in treating SSc showed conflicting results in skin and lung involvement but positive effects on myopathy [12]. The partially poor clinical response to glucocorticoid treatment in SSc might result from delays in its application, meaning that it cannot prevent the very early induction of vasculopathy and fibrosis arising as a result of inflammation [1, 13–15].

The European League Against Rheumatism (EULAR) Scleroderma Trials and Research Group has identified preliminary criteria for the very early diagnosis of systemic sclerosis (VEDOSS), which include the combination of Raynaud’s phenomenon, disease-specific auto-antibodies, typical nailfold capillaroscopic findings and “puffy fingers” [16, 17].

Recent developments in our understanding of the overlapping disease mechanisms of SSc and other autoimmune diseases, the inflammatory mechanisms involved in early SSc and the partial response of SSc treated with glucocorticoids suggest a possible treatment opportunity in patients with early SSc. The aim of this trial is to examine the effects of high-dose glucocorticoids on the inhibition of the inflammatory process in very early SSc.

## Methods/design

This 12-week, double-blind, randomised, placebo-controlled trial is under way (inclusion began on January 1, 2017, and is open until July 1, 2018) at the Department of Rheumatology of Radboud University Medical Centre in Nijmegen, The Netherlands.

### Objectives

The aim of this study is to analyse whether high-dose methylprednisolone has an effect on very early SSc in terms of nailfold capillaromicroscopy (NCM) abnormalities, SSc signs and symptoms, and inflammation biomarkers. This led to the following research questions.

### Primary objective

The purpose of this study was to determine the effect of short-term treatment with high-dose glucocorticoids on vasculopathic abnormalities, as measured by using NCM in patients with very early SSc.

### Secondary objectives

The key secondary objective is to determine the effects of the short-term treatment of SSc with high-dose glucocorticoids on changes in the following:

- the signs and symptoms of disease progression,
- inflammation biomarkers, and
- general health and utilities, as measured by standardised questionnaires.

### Study design rationale

In order to investigate the effect of high-dose methylprednisolone in very early SSc, we have designed a randomised, placebo-controlled, double-blind, mono-centre superiority trial with a primary endpoint of changes in nailfold capillary density after 12 weeks. Randomisation will be performed in blocks of six after the stratification of anti-centromere/anti-topoisomerase auto-antibodies, sex and age and there will be a 2:1 patient allocation to groups receiving intravenous methylprednisolone or placebo.

### Study outcome rationale

In SSc, the outcome measures of interventions are not extensively available. The most accepted outcome measure is a change in skin involvement, measured by using the modified Rodnan skin score (mRSS). However, in our study population of patients with very early SSc, the mRSS is 0 by definition at the outset. Nailfold capillary abnormalities have been identified as a symptom for diagnosing early SSc and are included in the American College of Rheumatology/EULAR (ACR/EULAR) classification criteria for SSc. Nailfold capillary abnormalities are evaluated by using NCM. The typical changes of the nailfold capillaries during SSc consist of the appearance of the following capillaroscopic hallmarks: the presence of enlarged or giant capillaries, haemorrhages, loss of capillaries, disorganisation of the microvascular array, and capillary ramifications. Semi-qualitative rating scores, especially of capillary density, have value in predicting the occurrence of organ involvement and mortality in SSc [18]. To date, the effect of immunosuppressant drugs on the nailfold capillaries in SSc has not been extensively studied. The most successful treatment of progressive SSc to date involved an autologous haematopoietic stem cell transplantation (ASCT), after which microcirculation estimated by using NCM was reported to be improved in separate studies [19, 20]. In patients who received ASCT, an improved capillary density was observed just 3 months after treatment and persisted during follow-ups over 24 months while no NCM modification was detected in patients who received cyclophosphamide [20]. In early SSc, the presence of giant capillaries is the dominant NCM feature, which can entail a decreased capillary density [21].

### Participants, intervention and outcomes

#### Inclusion criteria

Patients who meet the following criteria at randomisation are eligible for the study:

1. age of more than 18 years,
2. fulfil VEDOSS criteria [16]: Raynaud’s phenomenon AND positive test for anti-centromere or anti-topoisomerase antibodies AND nailfold capillaroscopic findings typical of SSc,
3. puffy fingers for less than 3 years, and
4. mRSS of 0.

#### Exclusion criteria

Patients who, at randomisation, meet any of the following criteria are not eligible for the study:

1. presence of acrosclerosis, acro-osteolysis or digital ulcers,
2. presence of anti-RNA polymerase III auto-antibodies,
3. previous systemic treatment for SSc, namely methotrexate, prednisone (>14 days in the previous 6 months), mycophenolate mofetyl or cyclophosphamide,
4. clinically significant internal organ involvement: diffusing capacity for carbon monoxide (DLCO) less than 80% predicted, vital capacity less than 70% predicted, renal dysfunction with glomerular filtration rate less than 60 mL/min, diastolic dysfunction more than grade 1 on echocardiography, pulmonary hypertension, or weight loss more than 10% in the last 6 months with unknown cause, and
5. contra-indications for methylprednisolone.

### Intervention

#### Investigational product/treatment

Eligible patients will be randomly assigned to groups, receiving either a daily infusion of methylprednisolone (1000 mg) or a placebo (physiologic salt solution identical in appearance) on three consecutive days in three consecutive months. The study drug and placebo will be provided by the local hospital pharmacy. Infusions will be given at the outpatient facilities of the rheumatology department.

#### Use of co-medication

All patients will receive prophylactic therapy with an angiotensin-converting enzyme inhibitor and a proton-pump inhibitor for the duration of the study. Treatment with other systemic therapies for SSc symptoms will be avoided whenever possible during the study and will be regarded as a protocol violation.

#### Follow-up treatment

After the 12-week assessment, patients will usually receive no treatment, as is the standard care in very early SSc. However, methotrexate (25 mg per week) can be started if a patient develops limited skin involvement but no significant internal organ involvement. Intravenous pulses of cyclophosphamide or mycophenolate mofetil can be started if a patient has rapidly evolving skin involvement or if signs of significant internal organ involvement are present or if both occur.

#### Escape treatment

In case of the significant worsening of symptoms (development of diffuse skin involvement, weight loss of more than 10%, or the occurrence of significant internal organ involvement), the treatment with methylprednisolone will be stopped and an appropriate treatment will be started in accordance with the EULAR treatment recommendations for SSc [22].

### Outcome measures

#### Primary outcome

In this study, the primary endpoint will be the change in capillary density between the baseline and after 12 weeks of treatment.

#### Secondary outcomes

The secondary outcomes (all compared between the baseline and week 12 or between the baseline and 1 year) of this study are (a change in):

- selected biomarkers: the interferon signature in peripheral blood cells, CXCL4, interleukin-1 beta (IL-1β), IL-6, tumor necrosis factor-alpha (TNF-α), ET-1, intercellular adhesion molecule 1 (ICAM-1) and vascular endothelial growth factor (VEGF) [3, 23–27];
- nailfold capillary changes other than capillary density and the presence of giant capillaries
- an mRSS; the presence of puffy fingers; the presence of synovitis; the presence of tendon friction rubs; fulfilling EULAR/ACR classification criteria for SSc [28];
- pulmonary function tests; the presence of interstitial lung disease; a suspicion of pulmonary hypertension; and
- physical function, general health and utilities as measured by the Scleroderma Health Assessment Questionnaire (SHAQ), 36-Item Short Form Health Survey (SF-36), EuroQOL five dimensions questionnaire (EQ5D) and Gastrointestinal Tract Questionnaire (GIT).

### Assessments

**Fig. 1:**
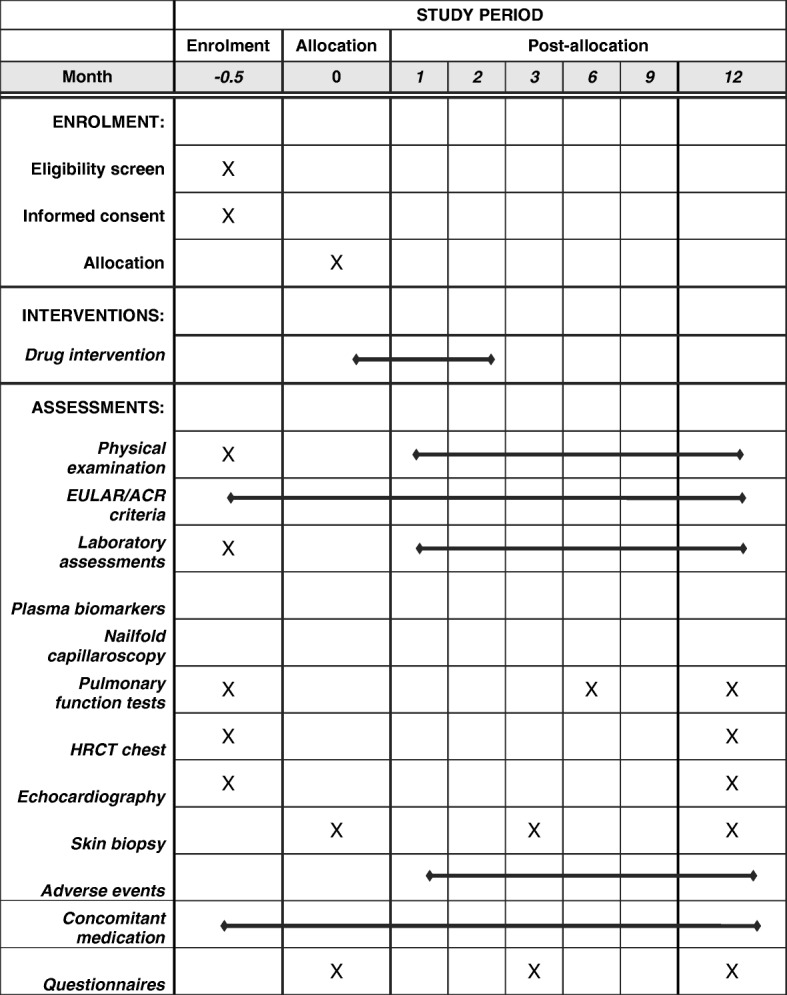
Study visits and procedures. Pulmonary function tests consisting of vital capacity, total lung capacity and carbon monoxide (CO) diffusion capacity. Plasma biomarkers consist of CXCL4, interleukin-1 beta (IL-1β), tumor necrosis factor alpha (TNFα), IL-6, ET-1, intercellular adhesion molecule 1 (ICAM-1) and vascular endothelial growth factor (VEGF). Abbreviation: *HRCT* high-resolution computer tomography

Regular visits are planned at the baseline and after months 1, 2, 3, 6, 9 and 12. See Fig. 1—according to the Additional file 1: SPIRIT (Standard Protocol Items: Recommendations for Interventional Trials) checklist—for an overview of all visits and assessments.

### Sample size

The effect of high-dose methylprednisolone on nailfold capillary changes and other outcomes in early SSc is unknown; therefore, the optimal size of a pilot trial is unknown. If the sample is too small, the estimate of the effect will be too uncertain. If the sample is too large, it may be judged as unnecessarily exposing participants to risks and burdens and therefore as being unethical. Furthermore, setting the sample size too high may lead to a preventable failure to reach the recruitment target. To minimise the risk for participants and maximise the information gained, we judged that the inclusion of 30 patients (20 in the treatment arm and 10 in the placebo arm) would be appropriate for this pilot study. When adopting the usual alpha of 0.05, power of 0.80, standard deviation of 0.9 and randomisation scheme of 2:1, a between-group difference of nearly 1.0 in the capillary density score thus would represent a statistically significant difference, using the standard sample size formula. In previous ASCT treatments for SSc, the change in capillary density over the first 12 weeks was 1.5 in the intervention group, and there was no change in the control group [20]. Patients are randomly assigned at a 2:1 ratio to maximise the information about the experimental intervention, which can be justified as the risks of the intervention are known and relatively low.

### Randomisation, concealment and blinding

Patients are randomly assigned in a 2:1 fashion to intravenous methylprednisolone or placebo in blocks of six, following stratification by either anti-centromere or anti-topoisomerase auto-antibodies, sex and age. Patients will be randomly assigned by using Castor, an online data collection tool for medical research. Randomisation will result in the allocation of a unique number to each patient. Physical examinations for efficacy outcomes are performed by a researcher who is blind to the occurrence of both mild and serious adverse events. The physicians who accompany the intervention and perform assessments for vital status and adverse events do not assess the efficacy outcomes. To maintain the overall quality and legitimacy of the clinical trial, the randomisation code can be broken only when knowledge of the actual treatment is absolutely necessary for further management of the patient or at the request of the safety monitoring committee.

### Statistical analysis

The data will be analysed primarily according to the intention-to-treat principle. Missing values due to participant drop-out are treated according to the last observation carried forward. A *P* value of 0.05 will be used to denote statistical significance for between-group differences in the primary outcome.

Between-group differences in the change in efficacy outcome variables between the baseline and week 12 will be analysed by using an analysis of covariance or logistical regression. Similarly, the ongoing changes to the continuous outcomes will be analysed between the baseline and weeks 4, 8 and 12 by using a repeated-measures analysis of variance.

Serious and minor adverse events will be tabulated by group, and depending on the occurrence, a time-to-event analysis may be used to analyse between-group differences in serious adverse events. The use of any immunosuppressive therapy between week 12 and the 1-year follow-up will be described for each of the two groups.

### Ethics and dissemination

The study has received ethical review board approval (number 2015–004613-24). Data on the recruitment, efficacy, safety, protocol updates and all aspects concerning good clinical practice are reviewed by the data safety monitoring board, which consists of an internal medicine physician, a pharmacist and an epidemiologist.

## Discussion

In designing this research protocol, we needed to address a number of clinical and methodological issues. First, patients who are still in the inflammatory phase of the disease are more likely to respond to treatment with glucocorticoids; therefore, the choice was made to include only patients who fulfil the VEDOSS criteria, but not the 2013 ACR/EULAR classification criteria, for SSc [28].

Second, treatment consisting of short pulses of methylprednisolone is often better tolerated than continuous high oral doses, while fewer long-term consequences are seen when using short methylprednisolone pulses. The choice of an intravenous infusion of high-dose (1000 mg) methylprednisolone was based on the treatment regimens in other auto-immune diseases, such as lupus nephritis and dermatomyositis, in cases of exacerbation or failing standard therapy or both [9, 10]. In these diseases, high-dose methylprednisolone has been shown to induce remission. The schedule of three consecutive days of treatment during three consecutive months was chosen because this method is generally used in severe autoimmune diseases.

Disease activity in SSc is measured mostly by mRSS and, in cases with pulmonary involvement, by pulmonary function results. However, to date, no biomarkers are available to follow-up the disease activity. The use of vasculopathy and inflammatory biomarkers, as well as clinical signs and symptoms, as endpoints in our study, enables us to meet the patient need for alternative markers on disease activity, as the known signs and symptoms of worsening prognosis are present in only a minority of patients.

This trial is the first to aim to treat very early SSc and is promising because it targets the early stages in the disease process using a relatively safe and inexpensive treatment known to be highly effective against inflammation. If it is possible to prevent significant clinical disease in patients with very early SSc by using a safe treatment, this will cause a paradigm shift in scleroderma care and research. The knowledge gained from this project will facilitate further research into the efficacy and safety of using high-dose glucocorticoids in SSc treatment and enable this treatment to progress through a pivotal phase III trial. We anticipate that, if successful, this project will enormously increase research efforts into the early treatment of SSc and decrease the cost of health care for these patients.

In this trial, patients with SSc receive treatment very early in the progression of their disease, which could change the long-term outcome of SSc. In current daily practice, patients so early in the disease course do not receive treatment, although they can be at risk for early escalation and internal organ involvement, reducing their prognosis. A trial to investigate the efficacy of treatment using high-dose glucocorticoids will provide us with the opportunity to change this disease course, reducing the disease burden of a portion of patients with SSc. If the treatment of very early SSc with high-dose glucocorticoids is effective, this will prelude a pivotal change in the treatment of SSc.

### Trial status

Open for inclusion.

## Additional file

Additional file 1: SPIRIT 2013 Checklist: Recommended items to address in a clinical trial protocol and related documents*. (DOC 121 kb)

## Abbreviations

ACR: American College of Rheumatology
ASCT: Autologous haematopoietic stem cell transplantation
EULAR: European League Against Rheumatism
mRSS: Modified Rodnan skin score
NCM: Nailfold capillaromicroscopy
SSc: Systemic sclerosis
VEDOSS: Very early diagnosis of systemic sclerosis
